# Regulatory Noncoding and Predicted Pathogenic Coding Variants of *CCR5* Predispose to Severe COVID-19

**DOI:** 10.3390/ijms22105372

**Published:** 2021-05-20

**Authors:** Sueva Cantalupo, Vito Alessandro Lasorsa, Roberta Russo, Immacolata Andolfo, Giuseppe D’Alterio, Barbara Eleni Rosato, Giulia Frisso, Pasquale Abete, Gian Marco Cassese, Giuseppe Servillo, Ivan Gentile, Carmelo Piscopo, Matteo Della Monica, Giuseppe Fiorentino, Giuseppe Russo, Pellegrino Cerino, Carlo Buonerba, Biancamaria Pierri, Massimo Zollo, Achille Iolascon, Mario Capasso

**Affiliations:** 1 Dipartimento di Medicina Molecolare e Biotecnologie Mediche, Università degli Studi di Napoli Federico II, 80136 Napoli, Italy; cantalupo@ceinge.unina.it (S.C.); lasorsa.alessandro@gmail.com (V.A.L.); russor@ceinge.unina.it (R.R.); immandolfo@gmail.com (I.A.); rosato.barbara@gmail.com (B.E.R.); gfrisso@unina.it (G.F.); massimo.zollo@unina.it (M.Z.); achille.iolascon@unina.it (A.I.); 2 CEINGE Biotecnologie Avanzate, 80145 Napoli, Italy; giusdalt95@gmail.com; 3 COVID Hospital, P.O.S. Anna e SS. Madonna della Neve di Boscotrecase, Ospedali Riuniti Area Vesuviana, 80042 Boscotrecase, Italy; abete2002@libero.it (P.A.); cassesegianmarco@gmail.com (G.M.C.); 4 Dipartimento di Neuroscienze e Scienze Riproduttive ed Odontostomatologiche, Università degli Studi di Napoli Federico II, 80131 Napoli, Italy; giuseppe.servillo@unina.it; 5 Dipartimento di Medicina Clinica e Chirurgia, Università degli Studi di Napoli Federico II, 80131 Napoli, Italy; ivan.gentile@unina.it; 6 Medical and Laboratory Genetics Unit, A.O.R.N. ‘Antonio Cardarelli’, 80131 Napoli, Italy; carmelo.piscopo@aocardarelli.it (C.P.); matteo.dellamonica@aocardarelli.it (M.D.M.); 7 AORN dei Colli Presidio Ospedaliero Cotugno, 80131 Napoli, Italy; giuseppefiorentino1@gmail.com; 8 Unità di Radiologia, Casa di Cura Villa dei Fiori, 80011 Acerra, Italy; dott-russo@libero.it; 9 Istituto Zooprofilattico Sperimentale del Mezzogiorno, 80055 Portici, Italy; strategia@izsmportici.it (P.C.); carlo.buonerba@izsmporticit.it (C.B.); biancamaria.pierri@izsmportici.it (B.P.); 10 Dipartimento di Medicina, Chirurgia e Odontoiatria “Scuola Medica Salernitana”, Università di Salerno, 84081 Baronissi, Italy

**Keywords:** *CCR5*, COVID-19, SARS-CoV-2, SNP, whole exome sequencing, GWAS

## Abstract

Genome-wide association studies (GWAS) found locus 3p21.31 associated with severe COVID-19. *CCR5* resides at the same locus and, given its known biological role in other infection diseases, we investigated if common noncoding and rare coding variants, affecting *CCR5*, can predispose to severe COVID-19. We combined single nucleotide polymorphisms (SNPs) that met the suggestive significance level (*P* ≤ 1 × 10^−5^) at the 3p21.31 locus in public GWAS datasets (6406 COVID-19 hospitalized patients and 902,088 controls) with gene expression data from 208 lung tissues, Hi-C, and Chip-seq data. Through whole exome sequencing (WES), we explored rare coding variants in 147 severe COVID-19 patients. We identified three SNPs (rs9845542, rs12639314, and rs35951367) associated with severe COVID-19 whose risk alleles correlated with low *CCR5* expression in lung tissues. The rs35951367 resided in a CTFC binding site that interacts with *CCR5* gene in lung tissues and was confirmed to be associated with severe COVID-19 in two independent datasets. We also identified a rare coding variant (rs34418657) associated with the risk of developing severe COVID-19. Our results suggest a biological role of *CCR5* in the progression of COVID-19 as common and rare genetic variants can increase the risk of developing severe COVID-19 by affecting the functions of *CCR5*.

## 1. Introduction

On 11 March 2020, World Health Organization (WHO) declared COVID-19 a pandemic. From the first case registered in December 2019 in Wuhan, over 160 million cases were registered in the world with over 3 million deaths (Access date: 19 May 2021, https://COVID19.who.int). These high numbers are mainly due to the strong power of SARS-CoV-2 (Severe Acute Respiratory Syndrome Coronavirus-2) transmissibility through the airways via human-to-human contact or droplet transfer [1]. Common symptoms of COVID-19 are fever or chills, cough, fatigue, muscle or muscle pain, headache, and loss of taste or smell [1]. In severe cases, the infection leads to breathing difficulties with dyspnea [2], and the most serious complication is the acute respiratory distress syndrome (ARDS), which is characterized by bilateral infiltrates, severe hypoxemia, and pulmonary edema [2]. Respiratory failure represents the principal cause of hospitalization of affected patients, which leads in the most cases to intubation with consequent mechanical ventilation [2,3]. The mortality rate caused by COVID-19 increases exponentially with age, which is the first risk factor [4,5]. The other factors that influence the course of the disease are pre-existent pathologies such as hypertension, diabetes, cardiovascular disease, and respiratory diseases [4,5]. Genetic predisposition may partly explain the severity of the phenotype in some COVID-19 patients.

A recent genome-wide association study (GWAS) has identified a genetic association between severe respiratory failure in COVID-19 hospitalized patients and the chromosome 3p21.31 [6] harboring multiple genes (*LC6A20*, *LZTFL1*, *CCR9*, *FYCO1*, *CXCR6*, *XCR1*, *CCR1*, *CCR3*, *CCR2* and *CCR5*) that could be functionally implicated in COVID-19 [6]. The genetic association of 3p21.31 locus with severe COVID-19 was recently confirmed by another independent GWAS, strengthening the role of this locus in disease susceptibility, and novel risk variants at chromosome 19p13.3, 12q24.13, and 21q22.1 were reported [7]. Another study has shown that inactivating rare mutations in genes belonging to the type I interferon pathway predispose to life-threatening COVID-19 pneumonia [8].

Once a disease-predisposing locus has been identified through a GWAS, in order to unravel the molecular basis of risk etiology, the main challenges to be faced are the identification of the causal genetic variants (i.e., those that actually contribute to the development of the disease) and the detection of the genes whose function is influenced by the same causal variants [9]. Therefore, post GWAS analyses of chromosome 3p21.31 are needed to unravel functional genetic variants and genes that may have a role in COVID-19 pathology.

The *CCR5* gene, located at the same locus risk 3p21.31, encodes a chemokine receptor and is expressed by immune system cell such as T cells and macrophages [10,11,12]. The deletion of 32 bp (*CCR5*-Delta32, rs333) in *CCR5*, which presents with an allelic frequency of about 10% in the Caucasian population, reduces susceptibility in Human Immunodeficiency Virus (HIV) infection in homozygosity [13,14,15]. CCR5 is also a target of the maraviroc drug used to treat patients with HIV [16]. *CCR5* seems to be of considerable importance in specific lung infections [17]; indeed, *CCR5* null mice show hyperacute inflammatory response following influenza A infection and mycobacterium tuberculosis, with an increased number of T lymphocyte and dendritic cell in the lung itself [18].

Based on these observations, we reasoned that functional genetic variants at identified GWAS risk 3p21.31 locus may affect the *CCR5* functions and predispose to severe disease. Most of the risk variants identified by GWASs localize to noncoding regions of DNA and exert their effects by influencing transcriptional output through multiple mechanisms. However, if *CCR5* has a key role in COVID-19 pathology, it is presumable that deleterious coding variants might be risk factors for disease as well.

Here, using summary statistics from GWAS meta-analysis of severe COVID-19 combined with expression Quantitative Traits Loci (eQTLs), chromosome conformation capture (Hi-C) data, and whole exome sequencing (WES) data, we aimed at identifying causal genetic variants that can specifically affect *CCR5* in order to understand the underlying mechanism of SARS-CoV-2 infection in patients with severe clinically manifestation of disease.

## 2. Results

### 2.1. CCR5 Is Highly Expressed in Lung and Bronchus

**Figure 1:**
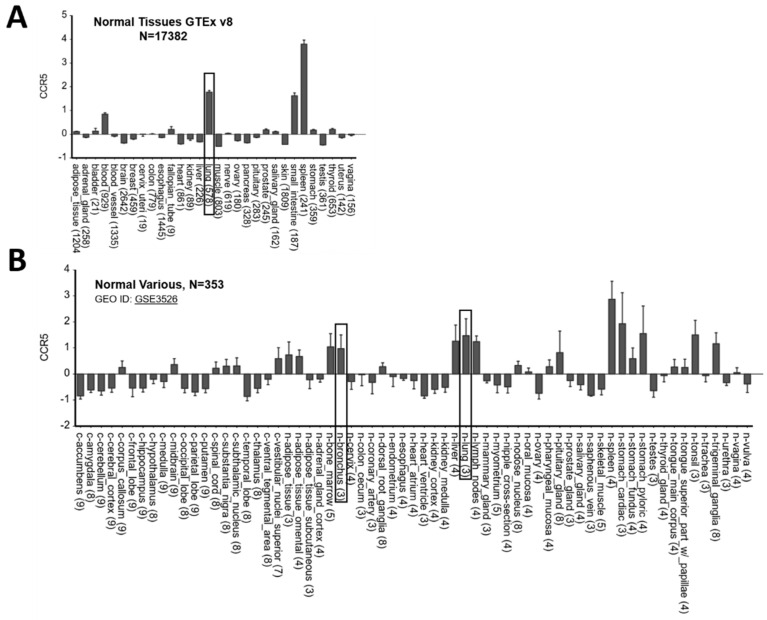
*CCR5* is highly expressed in lung and bronchus. (**A**) Gene expression data of 17,382 samples categorized in 30 different normal tissues obtained from GTEx database and (**B**) of 353 samples categorized in 69 different normal tissues obtained from Roth et al. study. The gray boxes highlight tissues from lung and bronchus. Data are plotted by using R2: Genomics Analysis and Visualization Platform (http://r2.amc.nl, accessed on 10 May 2021).

Starting from the hypothesis that *CCR5* has an important role in lung infection, we have analyzed *CCR5* expression in different tissues. *CCR5* was shown to be highly expressed in lung in two independent gene expression datasets of normal tissues profiled by GTEx consortium using RNA-Seq (Figure 1A) and by Roth et al. [19] using microarray (Figure 1B) technology. We also observed *CCR5* as highly expressed in bronchus (Figure 1B).

### 2.2. CCR5 Expression Is Affected by SNPs at Chromosome Risk Locus 3p21.31

**Figure 2:**
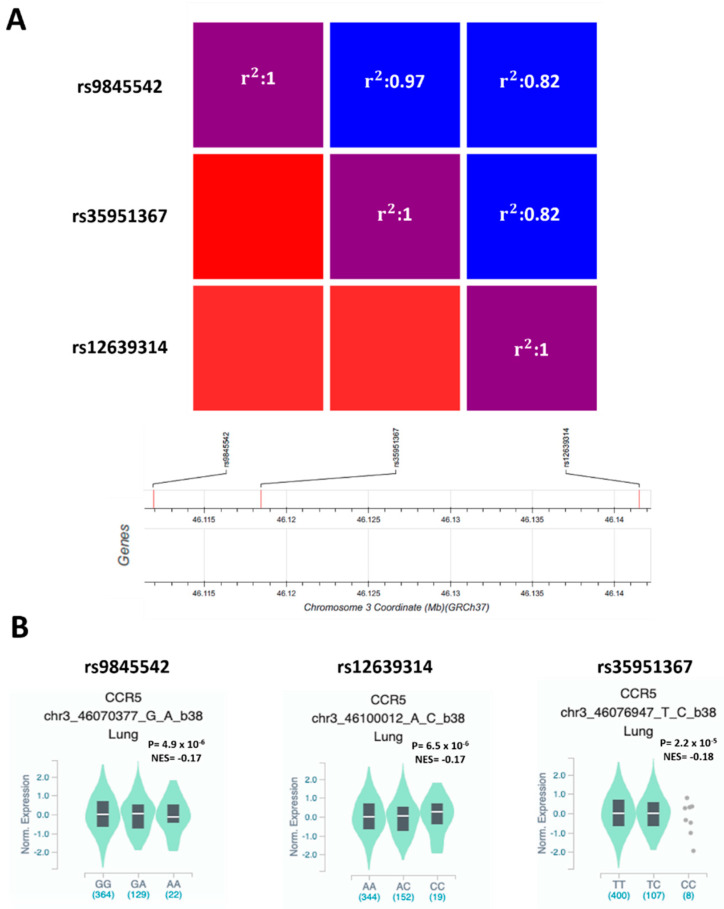
rs9845542, rs12639314, and rs35951367 SNPs are in LD in the European population, and all the minor alleles are associated with low expression of *CCR5* in lung. (**A**) Heatmap shows LD among the selected SNPs in the European population. (**B**) Violin plot showing the effect of eQTLs SNPs on *CCR5* expression in lung. NES: normalized effect size.

To verify if SNPs at the genome-wide associated locus 3p21.31 [6] may affect CCR5 functions, we used GWAS data of 6406 hospitalized COVID-19 patients and 902,088 controls of European origin from ‘The COVID-19 Host Genetics Initiative’ [20]. In the region on chromosome 3, which appears to be significantly associated with severe COVID-19 at the genome-wide level (https://www.covid19hg.org/results, accessed on 10 May 2021), we selected 52 SNPs with suggestive statistical significance (*P* ≤ 1 × 10^−5^) and that were eQTLs for *CCR5* in lung (208 samples from GTEx database) (Supplementary Table S1). The three SNPs with the highest eQTLs *p* values in lung were rs9845542 (*P* = 4.9 × 10^−6^), rs12639314 (*P* = 6.5 × 10^−6^), and rs35951367 (*P* = 2.2 × 10^−5^) (Supplementary Table S1). These three SNPs are in complete linkage disequilibrium (LD) with each other in European and South Asian populations (Figure 2A, Figure S1) but not in Africans, mixed Americans, and East Asians (Figure S1).

In particular, the frequency of minor allele of rs35951367 varies considerably according to the analyzed population: the minor allele C is more frequent in the European populations (non-Finnish: 16.1%) than other populations of non-European origin such as African, where the frequency is 10.1% or East and South Asian, where the allele frequency drops from 4.9% to 0% (Figure S2). However, all the minor alleles were associated with increased risk of developing severe COVID-19 (rs9845542 *P* = 4.2 × 10^−20^, OR = 1.33, 95% CI: 1.25–1.41; rs12639314 *P* = 7.1 × 10^−9^, OR = 1.23, 95% CI: 1.14–1.31; rs35951367 *P* = 3.2 × 10^−19^, OR = 1.32, 95% CI: 1.24–1.40) (Supplementary Table S1) and correlated with lower expression levels of *CCR5* in lung (Figure 2B).

**Figure 3:**
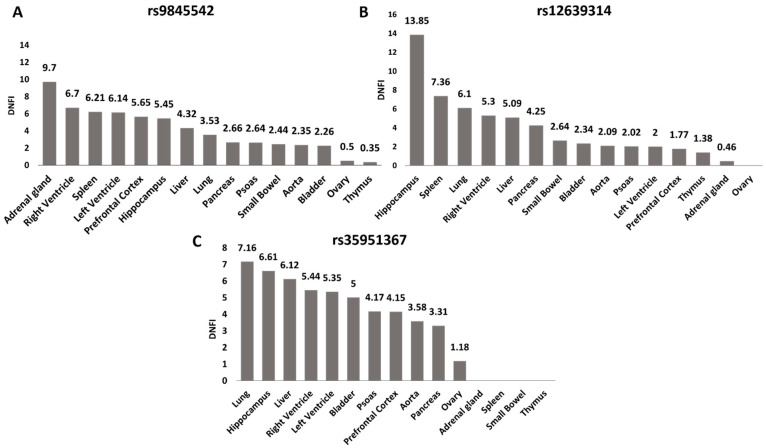
rs35951367 is located in the genomic region with the highest level of interaction with *CCR5* in lung. For all SNPs, the level of interaction with *CCR5* as Distance Normalized Interaction Frequency (DNIF) in all tissues is shown. (**A**) DNIF of rs9845542; (**B**) DNIF of rs12639314 and (**C**) DNIF for rs35951367. The highest levels of interaction with *CCR5* in lung were found for the (**C**) rs35951367 SNP. Data were retrieved from the 3DIV database (http://kobic.kr/3divv1/, accessed on 10 May 2021).

To further investigate the potential role of the selected SNPs in regulating *CCR5* expression, we used Hi-C data from 3DIV database [20] to explore the genomic interaction between the selected SNPs and our gene of interest. We compared Hi-C data of the three SNPs among all tissues present in the database (Figure 3A–C, Figure S3).

The rs35951367 SNP region showed the highest level of interaction with *CCR5* than the regions of the other three SNPs with a Distance Normalized Interaction Frequency (DNFI) of 7.16 in lung (Figure 3C, Figure S3, Supplementary Table S2). Notably, in lung, the value of interaction frequency of rs35951367-associated region was the highest value observed (Figure 3, Supplementary Table S2). When we repeated the same analysis using normal lung fibroblast cell line (IMR90), we again observed the strongest interaction between rs35951367-associated region and *CCR5* (Supplementary Table S3). Based on our results, we focused our attention on rs35951367, which was likely to be a regulatory SNP of *CCR5*.

**Figure 4:**
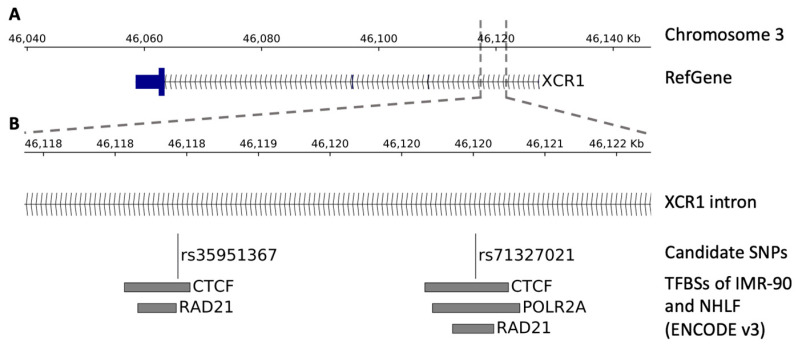
Genomic view of the region surrounding the SNPs. The figure shows the transcription factor binding sites (TFBSs) within the region surrounding the SNPs. (**A**) Genomic view of the SNP localization. (**B**) Zoom-in view showing, from top to bottom, the SNP localization, the candidate SNPs, and the TFBSs of the ENCODE catalogue (v3) belonging to the IMR-90 and NHLF lung cell lines.

Chip data from the ENCODE catalogue of two lung cell lines (IMR90 and NHLF) showed that rs35951367 falls in a CTCF binding region localized in the intron of *XCR1* gene (Figure 4A,B).

This SNP is an eQTL only for *CCR5* in lung and not for *XCR1* in lung or other tissues (Supplementary Table S4). By in-depth inspection analysis, closely located to rs35951367, we found the rs71327021 SNP that was previously excluded from our initial QTL analysis, as it did not pass the stringent levels of statistical correction. However, we verified that the rs71327021 SNP correlated with *CCR5* expression with a nominal level of significance (Figure S4A). Interestingly, this SNP is in LD with our candidate rs35951367 SNP (r^2^ = 0.76) and in addition, it falls in binding sites of CTFC (Figure 4B). However, analysis of Hi-C data showed the strongest interaction between rs71327021-associated region and *CCR5* was observed in spleen tissue and not in lung (Supplementary Table S5, Figure S4B).

### 2.3. Replication Studies

To confirm the association found in GWAS data for rs35951367, we conducted two replication studies. The first replication study was carried out using the genetic data from the 23andMe study [21]. In this cohort, including 1131 hospitalized COVID-19 patients, the minor allele C of rs35951367 was confirmed to be a risk factor for COVID-19 severe (OR = 1.41, *P* = 3.79 × 10^−6^); indeed, it was more frequent in cases (20%) than controls (15%). The second replication study was carried out using our Italian cohort of 202 hospitalized COVID-19 patients and 929 controls. Again, we confirmed the minor allele of rs35951367 as a risk factor for the more severe form of disease (OR = 1.307, *P* = 0.043) (Table 1).

As rs35951367 showed a different allelic frequency in different populations (Figure S2), we used GWAS data of 2244 critically ill patients with COVID-19 and UK biobank controls from the GenoMICC study [7] to validate the association of rs35951367 with severe COVID-19 phenotype in non-European populations. The association was not confirmed in Africans (*P* = 0.43, OR = 1.24, 95% CI 0.59–1.89), East Asians (*P* = 0.75, OR = 1.14, 95% CI: 0.22–2.06), and South Asians (*P* = 0.18, OR = 1.18, 95% CI: 0.89–1.47).

### 2.4. Coding Variants in CCR5

In parallel with investigation of *CCR5* locus in GWAS data, we conducted WES on 147 hospitalized COVID-19 patients of Southern Italy. In this WES dataset, we selected rare missense and INDEL variants (MAF < 0.01%) of *CCR5*. We found four variants in six patients (Table 2), and the rs34418657 variant was found in three patients (3/147, 2.04%).

The minor allele of rs34418657 showed a frequency of 0.03% in European (non-Finnish) population and was predicted to be pathogenic by seven different tools (Supplementary Table S6). Using the DUET tool [22], rs34418657 was predicted to destabilize the protein (ΔΔG = −0.893 Kcal/mol), suggesting its potential role as loss of function.

Motivated by these data, an additional 74 COVID-19 hospitalized samples and 1084 controls with Italian origin were typed using TaqMan assay for the rare variant rs34418657. Combining data from WES and TaqMan assay, we found that heterozygotes are more frequent in 221 patients with severe COVID-19 than in controls (P_GT_ = 0.027) (Table 3).

We also verified the frequency of 32 bp deletion (*CCR5*-Delta32, rs333), which was already associated with HIV resistance [15] using our 147 COVID-19 hospitalized patients and an Italian cohort of controls from public exomes data of 1095 Italian individuals (Network for Italian Genomes) [23]. No significant difference of frequency of *CCR5*-Delta32 was observed between controls (10.22%) and COVID-19 cases (10.20%), (*P* = 0.99; OR = 0.99, 95% CI: 0.57–1.73). To verify the association in GWAS dataset, we used the rs113341849 SNP in LD with rs333 (r^2^ = 0.97) and confirmed no association between *CCR5*-Delta32 (*P* = 0.11, OR = 0.92, 95% CI: 0.82–1.02).

## 3. Discussion

The clinical manifestations of COVID-19 are very heterogeneous; indeed, they can range from completely asymptomatic subjects up to patients who are hospitalized and undergo mechanical ventilation [1]. Numerous studies have focused on discovering the causes of this spectrum of phenotypes and on the evaluation of the presence of genetic differences in the predisposition to this pathology [24,25,26].

For example, two recent GWAS studies have demonstrated a genetic association between severe COVID-19 and the blood groups, where group O is a protective factor, and there are common variants at 3p21.31, 12q24.13, 19p13.2, 19p13.3 loci [6,7].

The 3p21.31 has been the first locus to be associated with the risk of developing aggressive forms of COVID-19 [6] and to be replicated in a different cohort of cases and controls [7]. The severe COVID-19 3p21.31 risk locus harbors several common variants and contains 17 known protein-coding genes including *LC6A20*, *LZTFL1*, *CCR9*, *FYCO1*, *CXCR6*, *XCR1*, *CCR1*, *CCR3*, *CCR2*, and *CCR5*. However, so far, causative variants and genes at this risk locus, influencing COVID-19 pathogenesis, remain to be investigated. Here, in order to study the potential biological role of the 3p21.31 risk locus, we focused our attention on the *CCR5* gene based on the motivations described below.

CCR5 is a chemokine receptor and is known in the literature for its role in infection pathogenesis, especially in HIV type 1 (HIV-1), where a deletion of 32 bp, present with an allele frequency of 12% in the northern Europe population, confers resistance at the HIV-1 infection in homozygous [13,27,28,29]. A study conducted on public genomes from Neanderthals to modern humans identified a total of 262 polymorphisms in the *CCR5* gene, with an SNP frequency per individual that ranged from 14 to 24 [30,31]. This high allelic heterogeneity is attributable to its role in the immune system as a result of co-evolution between humans and pathogens and is a peculiarity of only humans and not of other primates [31].

In leukocytes, CCR5 is a key receptor for chemotaxis from the bloodstream to the site of inflammation [32,33]; in particular, CCR5 is important for the rapid recruitment of memory CD8^+^ T cells to the lung airways during virus infection for limiting virus replication [34]. However, in addition to leukocytes, *CCR5* is variably expressed in many tissues [32,35,36,37,38]. Our in silico analysis shows that it is highly expressed in the lung from healthy individuals, suggesting its key biological role in the pathogenesis of COVID-19. Additional studies to verify the expression of *CCR5* in tissues from subjects with severe COVID-19 might further support its role in disease initiation and progression.

Starting from this evidence, we decided to investigate the CCR5 role in new SARS-CoV-2 infection using a dualistic approach: we first investigated the SNPs in noncoding regions, exploiting the freely available GWAS data, and then evaluated the coding variants obtained by WES of our cohort of severe cases.

We hypothesized that common variants at 3p21.31 may act as risk factors of severe COVID-19 by affecting the expression of *CCR5*. Combining public GWAS data with eQTLs data from the GTEx project, we have demonstrated that the minor allele of the rs35951367 SNP confers risk of severe COVID-19 and correlates with a lower expression of *CCR5* in lung. In accord with our results, a preprint paper reports a transcription-wide association study that found *CCR5* expression to be regulated by variants at 3p21.31 only in lung tissue [39].

Interestingly, Hi-C data obtained in lung tissue and cell lines showed that the rs35951367 SNP is located in a DNA region interacting with *CCR5* and marked by CTCF binding in two lung cell lines (IMR90 and NHLF). CTCF can function as a transcriptional activator, a repressor, or an insulator protein, blocking the communication between enhancers and promoters [40]. It plays a relevant role in contributing to genome organization and gene expression [40,41]. Therefore, our data suggest that the rs35951367 SNP may functionally act by altering topological domains and thus the normal transcriptional program of the *CCR5* gene. In vitro experimental studies are needed to confirm these findings and, in addition, to further genetic studies of variants already found to be associated with the different expression of *CCR5* [42,43,44].

Very close to rs35951367, we found the rs71327021 SNP, which also falls into the CTCF-binding region in lung cell lines, but it is not an eQTL for *CCR5* in the lungs. However, the rs71327021 SNP may be a good candidate to be tested in future genetic and functional studies.

Replication study is the golden standard for validating genome-wide association findings; thus, we used the genetic data from the 23andMe study and typed the rs35951367 SNP by TaqMan assay in cases and controls from Southern Italy; in both cohorts, the genetic association was confirmed. However, the same variant was not found to be associated with severity of COVID-19 illness in non-European subjects such as Africans and Asians, which was likely because of the lower frequency of the variant observed in these populations with respect to Europeans. So, we can speculate that this genetic risk factor may be one of the causes linked to the different rate of mortality observed across the different populations. Future studies will help to better define the effect of genetic variants at the *CCR5* locus on the clinical subgroups of COVID-19 disease—for instance, performing association analyses on patients stratified by disease aggressiveness.

Together, our findings provide evidence that common functional SNPs can predispose to clinically severe COVID-19 by long-range interaction with *CCR5* and that this gene can play a relevant role in the pathogenesis of SARS-CoV-2 infection.

In accordance with our findings, it was observed that the deficiency of CCR5 in West Nile virus infection, which shares with COVID-19 the multitude of phenotypes ranging from the subclinical to the most severe cases, represents a risk factor to develop severe symptoms [45]. Furthermore, in vivo studies indicate that mice CCR5^−/−^, infected with West Nile virus, have an elevated mortality rate and viral loads compared with wild-type mice [46].

In addition, the lung appears to be a particularly sensitive tissue to low *CCR5* expression as demonstrated in studies of influenza A virus infection, where *CCR5* deficiency is associated with poor outcome and with an increased mortality [18,47]. Notably, null mice of CCR5 show an accelerated macrophage accumulation in lungs with an increased expression of RANTES, MCP-1, and IP-10 [18]. The latter is one of three pro-inflammatory cytokines that correlate with rapid disease progression in hospitalized COVID-19 patients [48]. The above-mentioned scientific evidence is in agreement with our findings according to which a reduced expression of *CCR5* in the lungs can promote COVID-19 progression.

Since *CCR5* is known in the literature for its numerous polymorphisms, we decided to also investigate if there are rare pathogenic coding variants associated with the severe COVID-19 phenotype. In our cohort of hospitalized patients profiled by WES, we found an association of a rare variant in exon 3 (rs34418657) predicted to destabilize the protein product. This variant has been already described in association study with unexplained recurrent pregnancy loss. This association was explained, as CCR5 is necessary for maternal–fetal tolerance, and rs34418657 was predicted to destabilize the protein [49]. Our observations further suggest that genetic alterations that reduce the CCR5 function predispose to COVID-19 progression and highlight once again the key role of *CCR5* in SARS-CoV-2 infection.

Conversely, the *CCR5*-Delta32 (rs333) common coding variants, known to protect from HIV infection [50], seem to be not associated with a minor risk of developing severe forms of COVID-19, as indicated by both our WES and GWAS results. These data provide evidence that rare and common coding variants of *CCR5* can have different and independent risk effects on the onset and progression of infectious diseases.

In conclusion, we demonstrate that common noncoding regulatory variants at the previously identified 3p21.31 risk locus and rare pathogenic coding variants in *CCR5* can contribute to severe clinical manifestations of COVID-19 by affecting CCR5 functions. Therefore, these findings suggest that *CCR5* is a good candidate to be experimentally studied for unravelling the biological basis of COVID-19 progression and for developing novel therapeutic interventions.

## 4. Materials and Methods

### 4.1. GWAS Data

Summary statistics (P-value, and effect size) were obtained from the GWAS dataset “B2_ALL_eur_leave_23andme”, deposited in the fourth round of GWAS meta-analysis of COVID-19 Host Genetics Initiative website (www.COVID19hg.org, accessed on 10 May 2021) [51], available since 20 October 2020. The GWAS dataset includes 6406 hospitalized COVID-19 patients and 902,088 members of the general population with European genetic ancestry and does not include 23andMe cohort. The COVID-19 Host Genetics Initiative was a global initiative to elucidate the role of host genetic factors in the susceptibility and severity of the SARS-CoV-2 virus pandemic.

### 4.2. Replication Study of rs35951367 SNP

To validate the association between rs35951367 and a severe form of COVID-19 disease, we use two independent cohorts of cases and controls.

The first cohort data were obtained from ‘23andMe COVID-19 Study’ [21]. This study includes 1131 COVID-19 patients reported to have a positive test and hospitalization following SARS-CoV-2 infection. Participants answered web questions about symptoms of cold or flu-like illnesses, COVID-19 diagnosis and testing, hospitalization, and severity of illness. Participants provided informed consent and participated in the research online, under a protocol approved by the external AAHRPP-accredited IRB, Ethical & Independent Review Services (E&I Review). Participants were included in the analysis on the basis of consent status as checked at the time that data analyses were initiated. The full GWAS summary statistics for the 23andMe discovery dataset will be made available through 23andMe to qualified researchers under an agreement with 23andMe that protects the privacy of the 23andMe participants. Please visit https://research.23andme.com/collaborate/#dataset-access/ (accessed on 10 May 2021) for more information and to apply to access the data.

The second cohort included 929 healthy controls and 212 hospitalized COVID-19 collected from our research group that was genotyped by TaqMan^®^ SNP Genotyping Assay for rs34418657 SNP (Applied Biosystems by Thermo Fisher Scientific, Waltham, MA, USA). Twenty DNA samples failed the genotyping. The healthy controls cohort was composed of 39 subjects that resulted to be positive for anti-SARS-CoV2-IgG antibodies but without any symptoms and 890 healthy controls recruited before the COVID-19 pandemic. Hardy–Weinberg equilibrium was evaluated using the goodness-of-fit chi-square test in control and cases subjects (*P* > 0.05). Clinical characteristics of cases and controls are reported in Supplementary Table S7.

Exomes of 1095 Italian individuals were retrieved from the web database Network for Italian Genomes (NIG) (http://nigdb.cineca.it/index.php, accessed on 10 May 2021) [23] for evaluating the association of rs333 in our cohort of patients.

### 4.3. In Silico Analysis

Allele and genotype frequencies were obtained from gnomAD (https://gnomad.broadinstitute.org/, accessed on 10 May 2021) [47]; eQTLs analysis was performed by using public data from Genotype-Tissue Expression (GTEx) Portal (https://gtexportal.org/home, accessed on 10 May 2021). ChIP data of transcription factor in two lung cell lines (IMR90 and NHLF) was obtained from ENCODE catalogue [48,49]. *CCR5* gene expression was obtained from NCBI Gene Expression Omnibus (GEO) Database [50] and plotted using R2: Genomics Analysis and Visualization Platform (http://r2.amc.nl, accessed on 10 May 2021). 3DIV was used for the analysis of Hi-C data (http://kobic.kr/3divv1/, accessed on 10 May 2021) [21]. DUET was used to evaluate effect of missense variants on CCR5 protein (http://biosig.unimelb.edu.au/duet/stability, accessed on 10 May 2021) [22].

### 4.4. Whole Exome Sequencing

We collected COVID-19 samples from several hospitals in Southern Italy: Azienda ospedaliera specialistica dei colli, Monaldi-Cotugno; Azienda Ospedaliera di Rilievo Nazionale Antonio Cardarelli, P.O. Ospedale Boscotrecase, Villa dei Fiori S.r.l.- Acerra. The genomic DNA of COVID-19 patients was extracted from peripheral blood using a Maxwell^®^ RSC Blood DNA Kit (Promega, Madison, WI, USA), and DNA concentration and purity were evaluated using a NanoDrop™ 8000 Spectrophotometer. For library preparation, we measured the DNA concentration of 147 COVID-19 patients with a Qubit^®^ DNA Assay Kit in Qubit^®^ 2.0 Fluorometer (Life Technologies, Carlsbad, CA, USA), and 1.0 μg of DNA was used for library preparation. Genomic DNA was sonicated to generate 180–250 bp fragments and then end-polished, A-tailed, and ligated with the full-length adapter with further PCR amplification. PCR products were purified using AMPure XP system (Beckman Coulter, Brea, CA, USA) and quantified using the Agilent high sensitivity DNA assay on the Agilent Bioanalyzer 2100 system. The whole exome was captured with Agilent SureSelect Human All Exome V6 (Agilent Tecnologies, Santa Clara, CA, USA), and the sequencing was performed on an Illumina NovaSeq 6000 Systems.

### 4.5. Bioinformatic Analysis of Sequencing Data

The paired end (2 × 150 bp) sequencing returned an average of 42 million raw reads per sample. After removing sequencing artifacts, we retained, on average, the 98.35% of raw reads. The percentage of bases with quality scores above 20 and above 30 (Q20 and Q30) was 97.7% and 93.8%, respectively. The cleaned reads were aligned versus the reference genome (GRCh37/hg19) and mapping BAM files were obtained with BWA-mem (version 0.7.17) and SAMTools (version 1.8). On average, 99.89% of reads were mapped and 20.47% of duplicate reads were removed with Picard (version 2.18.9). We covered 99.63% of the target regions, and the average sequencing depth on target was 149.06x. Most (95.08%) of the target regions were covered with at least 20 reads, which were sufficient for reliable variant calls. SNVs and small insertions and deletions (INDELs) were detected with GATK HaplotypeCaller, and the functional annotation of variants was performed with ANNOVAR. We obtained 189,625 raw SNVs and 32,089 raw INDELs per sample. We excluded off-target variants (e.g., intergenic, intronic, etc.) and single nucleotide polymorphisms (SNPs) with allele frequencies greater than 1% in non-Finnish European populations of the 1000 Genomes Project, ExAC (v3) and GnomAD (v2.1.1) databases. To remove possible false positives, we also discarded variants falling in genomic duplicated regions. Then, the set of exonic variants was filtered to remove synonymous SNVs.

### 4.6. Association Study of the Coding Rare Variant rs34418657

In order to assess the association of the rare variant rs34418657, identified through WES, we genotyped by TaqMan^®^ SNP Genotyping Assay (Applied Biosystems by Thermo Fisher Scientific, Waltham, MA, USA) 1084 healthy controls and an additional 74 COVID-19 hospitalized patients not analyzed by WES. Fifty controls and one COVID-19 patient failed the genotyping. Healthy controls cohort was composed of 379 people that were shown to be positive for anti-SARS-CoV2-IgG antibodies but without any symptoms and 705 healthy controls recruited before the COVID-19 pandemic. A higher number of healthy controls with respect to common variant rs35951367 was typed in order to gain more statistical power to detect significant genetic differences since the rs34418657 variant is very rare in the general population. Hardy–Weinberg equilibrium was evaluated using the goodness-of-fit chi-square test in control and case subjects (*P* > 0.05). Clinical characteristics of this large cohort of controls are also reported in Supplementary Table S7.

DNA samples from patients were obtained after they signed informed consent and according to the Declaration of Helsinki. Local university ethical committees approved the study.

## Figures and Tables

**Table 1:** Association of common regulatory variant rs35951367 with severe COVID-19 in the Italian cohort.

	Italian Cases *n* = 202		Italian Controls *n* = 929		*P*	OR (CI: 95%)
rs35951367	n	%	n	%		
Genotype						
TT	120	59	625	67	-	
TC	72	36	266	29	0.038	1.410 (1.018–1.952)
CC	10	5	38	4	0.39	1.371 (0.665–2.826)
Allele						
T	312	77	1516	82	-	
C	92	23	342	18	0.043	1.307 (1.007–1.696)
Dominant						
TT/TC	192	95	891	96	-	
CC	10	5	38	4	0.39	1.371 (0.665–2.826)
Recessive						
TT	120	59	625	67	-	
TC/CC	82	41	304	33	0.032	1.405 (1.028–1.920)

OR: odd ratio; CI: confidence interval.

**Table 2:** Coding variants found in *CCR5*.

Sample ID	Chr Position (hg19)	Ref	Alt	Func. Ref Gene	AA Change (CCR5: NM_000579)	SNP	CADD
R- 16	chr3:46415061	G	A	nonsynonymous SNV	exon3: c. G668A: p. R223Q	rs1800452	22.5
R- 68	chr3:46414611	C	T	nonsynonymous SNV	exon3: c. C218T: p. A73V	rs56198941	31
R- 135	chr3:46415073-46415075	AGA	-	nonframeshift deletion	exon3: c. 680_682del: p. K229del	rs774845977	/
R- 30	chr3:46414784	G	T	nonsynonymous SNV	exon3: c. G391T: p. V131F	rs34418657	29.8
R- 60	chr3:46414784	G	T	nonsynonymous SNV	exon3: c. G391T: p. V131F	rs34418657	29.8
R- 72	chr3:46414784	G	T	nonsynonymous SNV	exon3: c. G391T: p. V131F	rs34418657	29.8

Chr: chromosome; Ref: reference and non-effect allele; Alt: alternative and effect allele.

**Table 3:** Association of rare coding variant rs34418657 with severe COVID-19 in the Italian cohort.

	Italian Cases *n* = 221		Italian Controls *n* = 1084		*P*	OR (CI: 95%)
rs34418657	n	%	n	%		
Genotype						
GG	217	98	1079	99.5	-	
GT	4	2	5	0.5	0.027	3.978 (1.060–14.933)
TT	0	-	0	-		
Allele						
G	438	99	2163	99.8	-	
T	4	1	5	0.2	0.116	3.951 (1.057–14.771)
Recessive						
GG	217	98.2	1079	99.5	-	
GT/TT	4	1.8	5	0.5	0.027	3.978 (1.060–14.933)

OR: odds ratio; CI: confidence interval.

## Data Availability

The GWAS dataset ‘B2_ALL_eur_leave_23andme’ is accessible at www.COVID19hg.org (accessed on 10 May 2021). The full GWAS summary statistics for the 23andMe discovery dataset will be made available through 23andMe to qualified researchers under an agreement with 23andMe that protects the privacy of the 23andMe participants at https://research.23andme.com/collaborate/#dataset-access/ (accessed on 10 May 2021) for more information and to apply to access the data. Exomes of healthy Italian individuals are accessible via the database Network for Italian Genomes (NIG) (http://nigdb.cineca.it/index.php, accessed on 20 March 2021).

## Supplementary Materials

The following are available online at https://www.mdpi.com/article/10.3390/ijms22105372/s1. Figure S1: Top *CCR5* eQTLs SNPs in lung are not in LD in African and AMR populations. Figure S2: Minor allele frequencies of the selected SNPs in different populations. Figure S3: rs35951367 shows the highest level of interaction with *CCR5* in lung. Figure S4: Minor allele of rs71327021 was associated with low expression of *CCR5* in lung, but this last one is not tissue, where interaction with *CCR5* is stronger. Table S1: eQTL SNPs for *CCR5* in lung. Table S2: Results of analysis of Hi-C data of rs35951367 region in lung. Table S3: Results of analysis of Hi-C data for rs35951367 region in lung cell line (IMR90). Table S4: Results of eQTLs analysis of rs35951367 in all tissues. Table S5: Results of the analysis of Hi-C data for the rs71327021 region in different normal tissues. Table S6: Rare coding variants of the *CCR5* gene in 147 COVID-19 hospitalized cases. Table S7: Clinical characteristics of COVID-19 hospitalized patients and healthy controls.
