# Supplementary material for: Regulatory Noncoding and Predicted Pathogenic Coding Variants of CCR5 Predispose to Severe COVID-19
Source: Int J Mol Sci. 2021 May 20;22(10):5372. doi: 10.3390/ijms22105372 (PMC8161088; doi:10.3390/ijms22105372)
Supplement: Supplementary file 1 [file ijms-22-05372-s001.zip › ijms-1214236-supplementary.pdf]

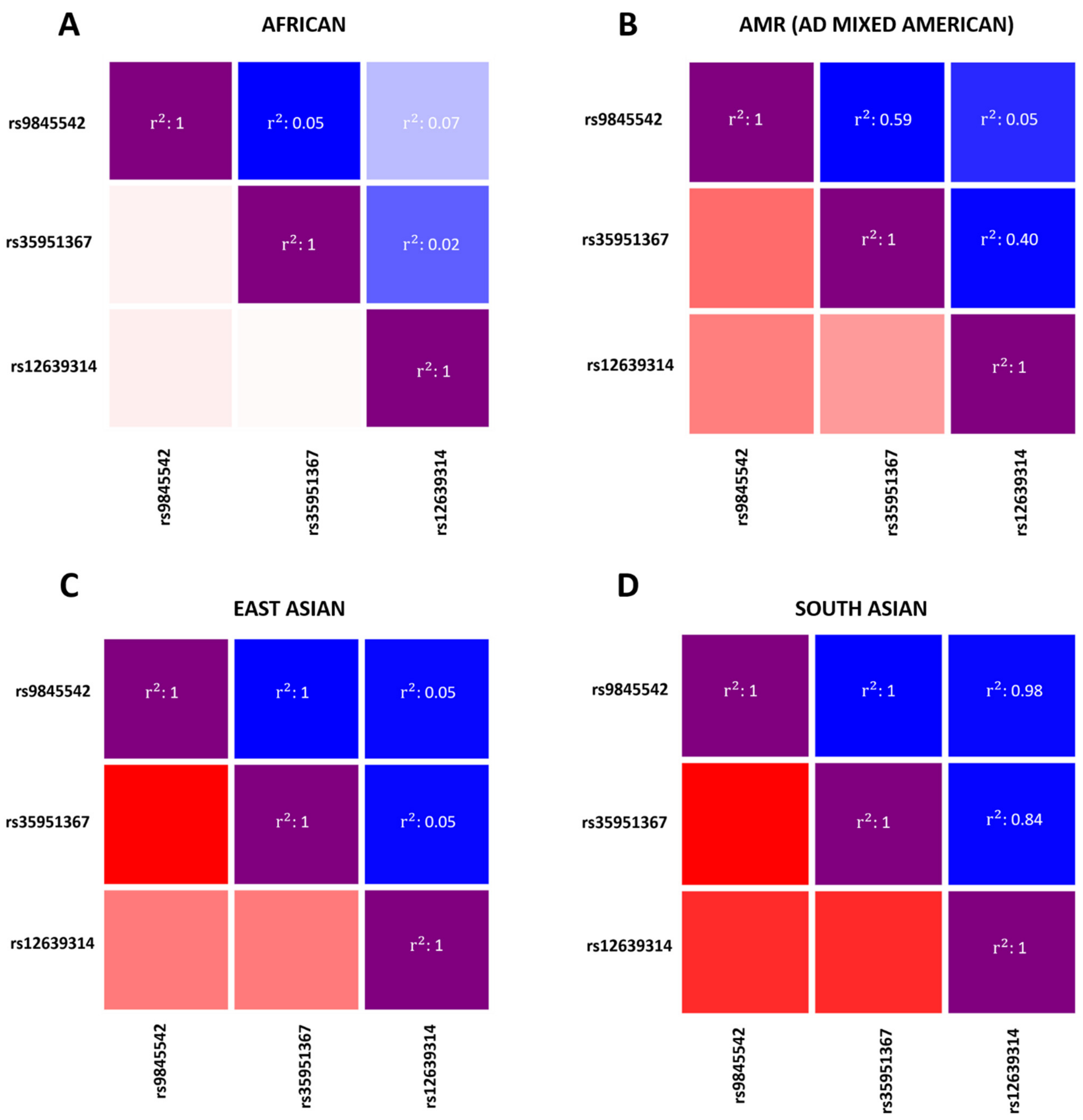

Figure S1. Top *CCR5* eQTLs SNPs in lung are not in LD in African (**A**) and AMR populations (**B**). In Asian population, our selected SNPs are in LD only in South Asian (**D**) while in East Asian (**C**), only rs35951367 and rs9845542 are in LD with (r^2=1). The D' and r^2 data are computed by using the web tool LD-link (https://ldlink.nci.nih.gov/?tab=home).

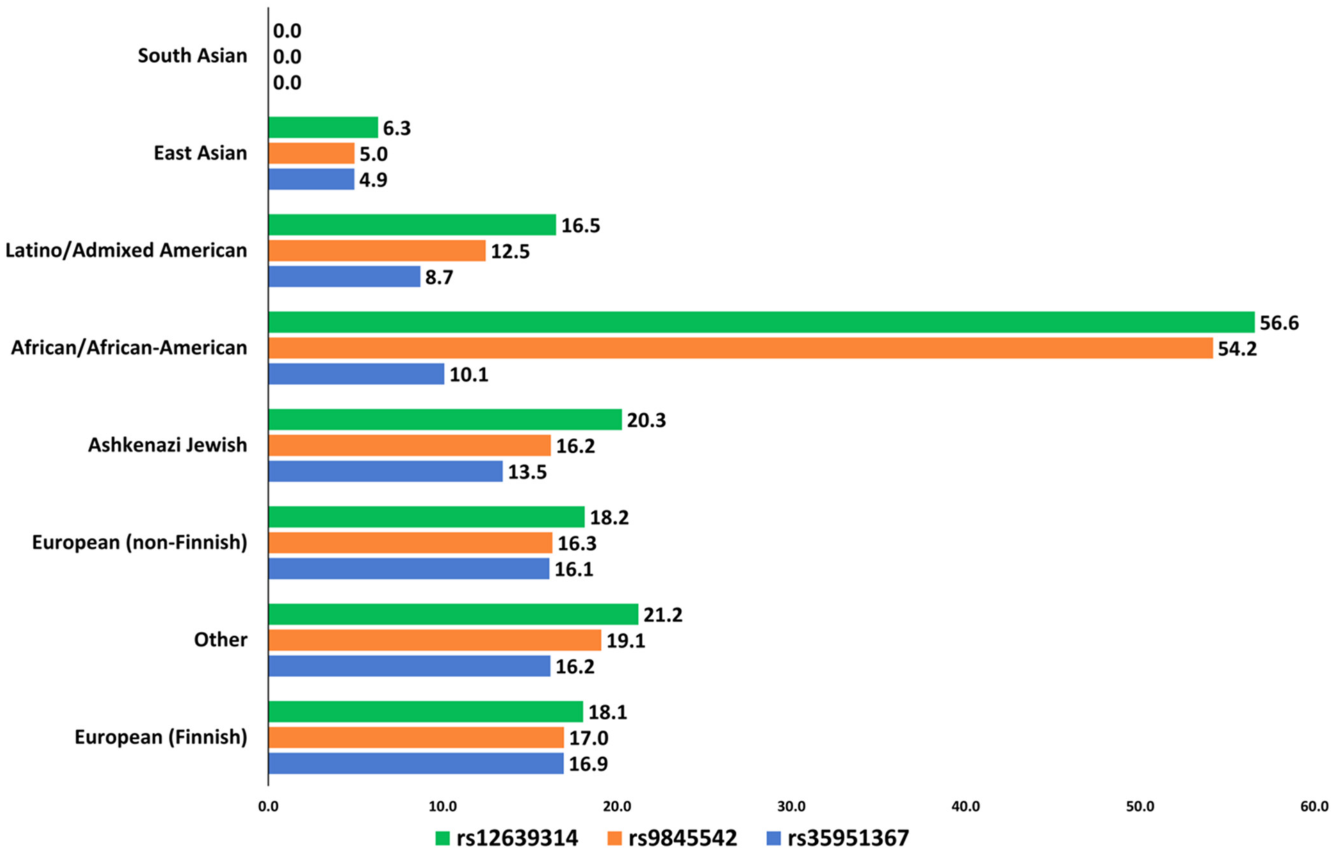

Figure S2. Minor allele frequencies of the selected SNPs in different populations. The graph shows the frequency of minor allele (expressed as percentage) of rs9845542, rs12639314 and rs35951367 in different populations. The minor allele C of rs35951367 is more frequent in European populations than in non-European ones. Frequency data was taken from gnomAD browser (https://gnomad.broadinstitute.org/).

A

rs9845542

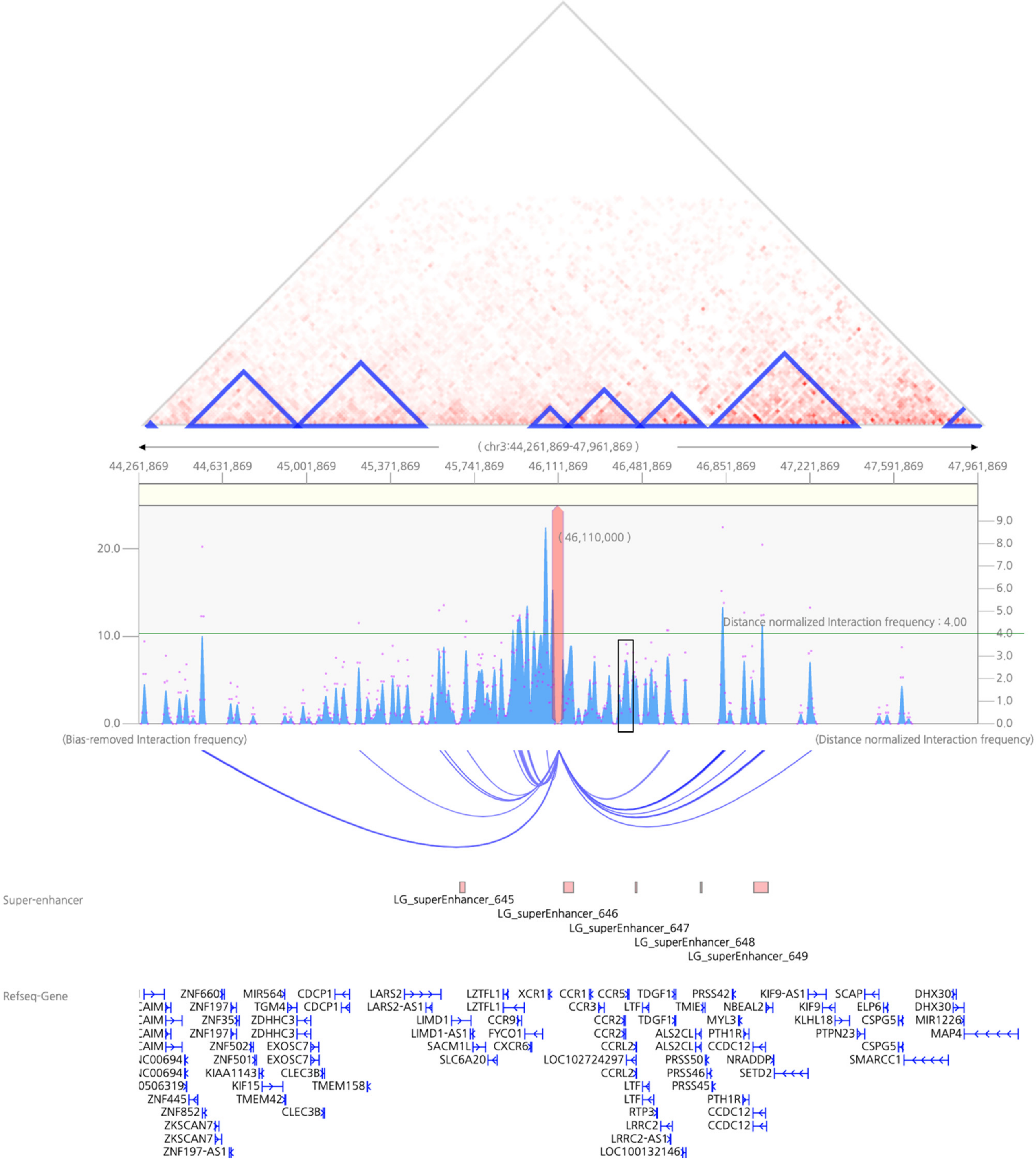

(chr3:44,261,869-47,961,869)
44,261,869
44,631,869
45,001,869
45,371,869
45,741,869
46,111,869
46,481,869
46,851,869
47,221,869
47,591,869
47,961,869
(46,110,000)
20.0
10.0
0.0
9.0
8.0
7.0
6.0
5.0
4.0
3.0
2.0
1.0
0.0
Distance normalized Interaction frequency : 4.00
(Bias-removed Interaction frequency)
(Distance normalized Interaction frequency)
Super-enhancer
LG_superEnhancer_645
LG_superEnhancer_646
LG_superEnhancer_647
LG_superEnhancer_648
LG_superEnhancer_649
Refseq-Gene
ZNF660
ZNF197
ZNF35
ZNF197
ZNF502
ZNF501
KIAA1143
KIF15
TMEM42
ZNF445
ZNF852
ZKSCAN7
ZKSCAN7
ZNF197-AS1
MIR564
TGM4
ZDHHC3
ZDHHC3
EXOSC7
EXOSC7
CLEC3B
CLEC3B
CDCP1
CDCP1
TMEM158
LARS2
LARS2-AS1
LIMD1
LIMD1-AS1
SACM1L
SLC6A20
LZTFL1
LZTFL1
CCR9
FYCO1
CXCR6
XCR1
CCR1
CCR3
CCR5
CCR2
CCR2
CCRL2
CCRL2
LOC102724297
LTF
LTF
LTF
RTP3
LRRC2
LRRC2-AS1
LOC100132146
TDGF1
TDGF1
ALS2CL
ALS2CL
PRSS50
PRSS46
PRSS45
TMIE
MYL3
PTH1R
PTH1R
CCDC12
CCDC12
CCDC12
PRSS42
NBEAL2
NRADDP
SETD2
KIF9-AS1
KIF9
KLHL18
PTPN23
SCAP
ELP6
CSPG5
CSPG5
SMARCC1
DHX30
DHX30
MIR1226
MAP4

B **rs12639314**

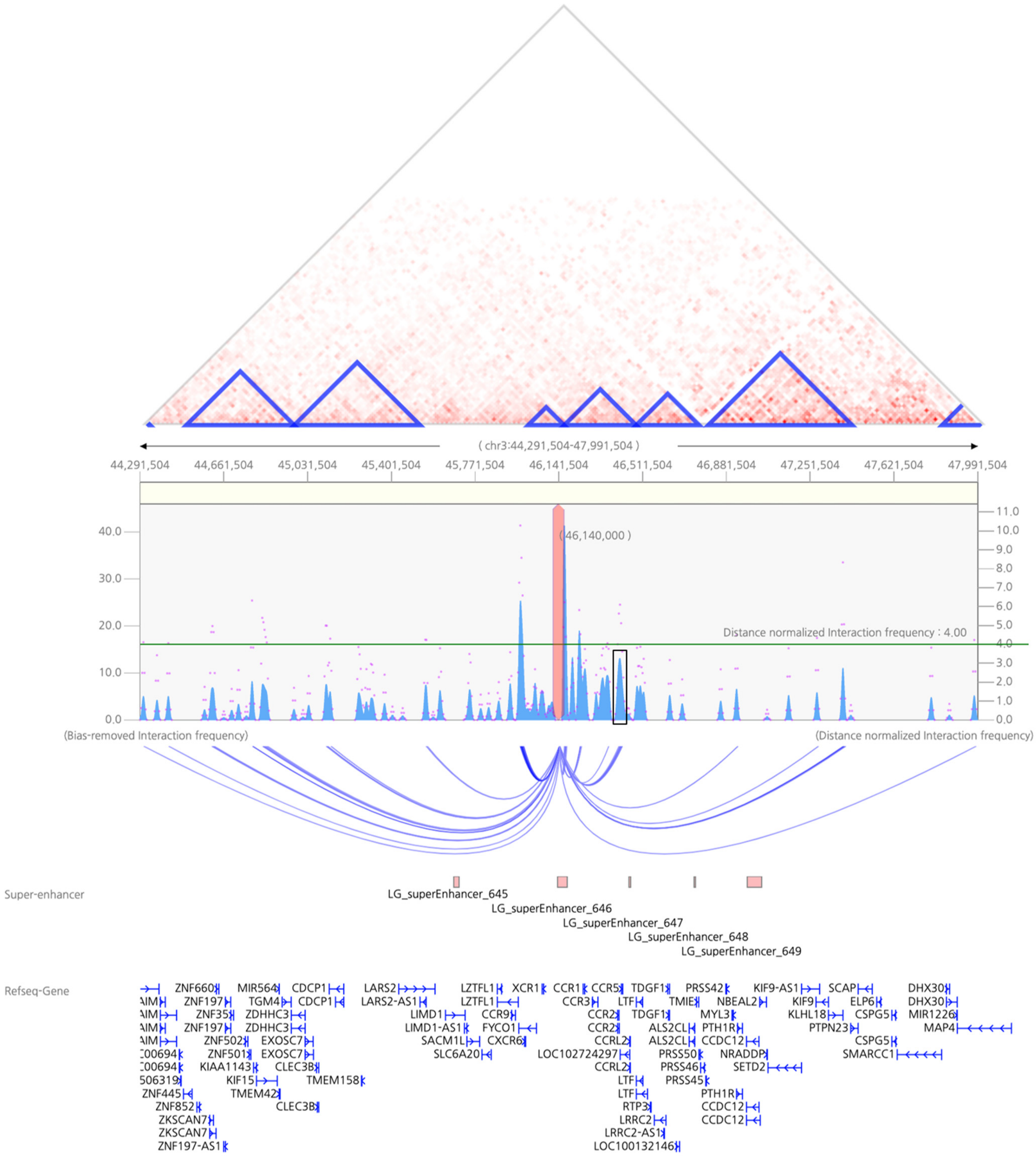

C **rs35951367**

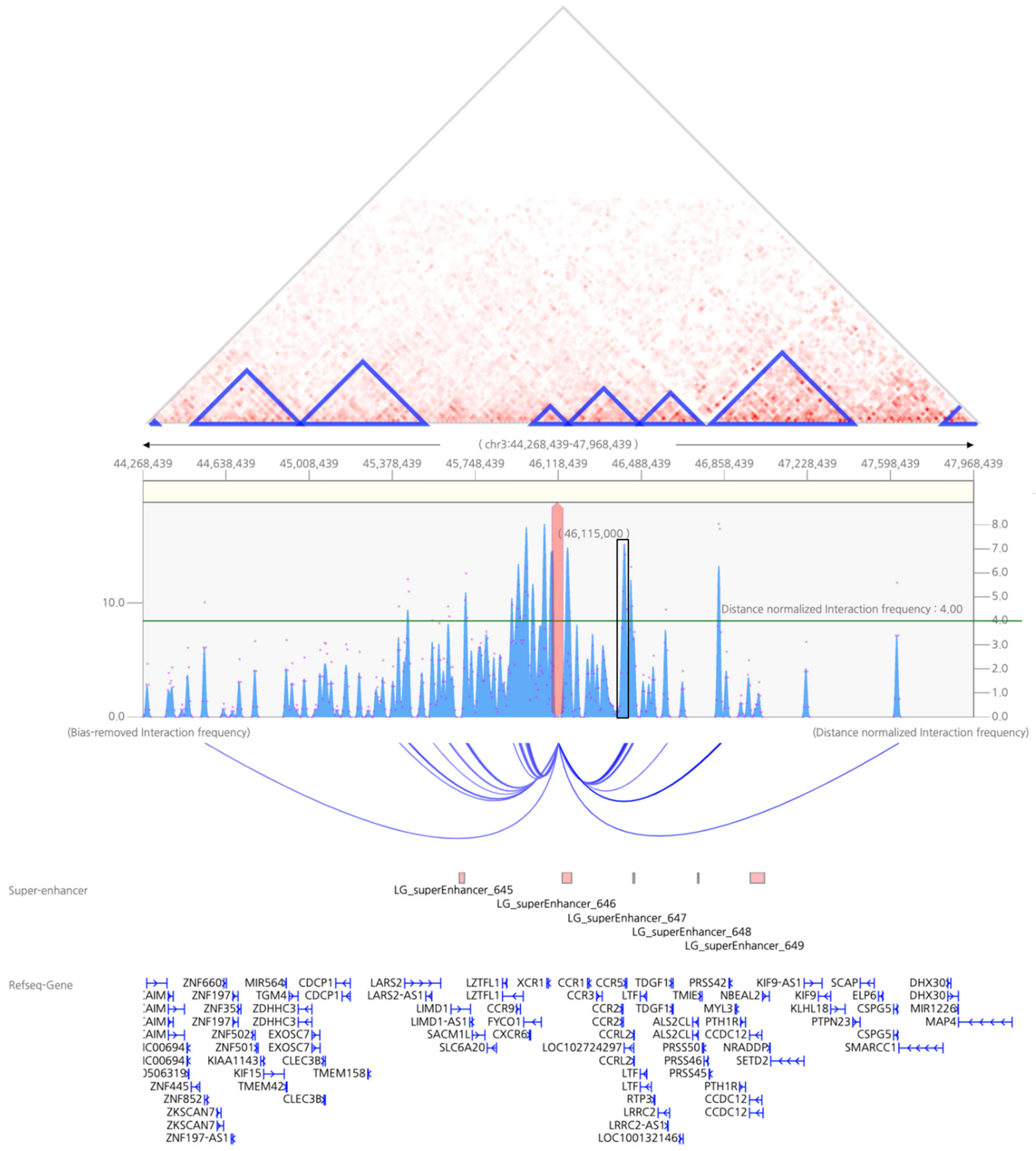

Figure S3. rs35951367 shows the highest level of interaction with *CCR5* in lung**.** Figures show the Hi-C data of 3 selected SNPs. Black boxes show the interaction value, expressed as Distance Normalized Interaction Frequency (DNFI), between the selected SNPs and *CCR5* in lung. The cut-off value is defined by the green line. Data were obtained from 3DIV (http://kobic.kr/3div/).

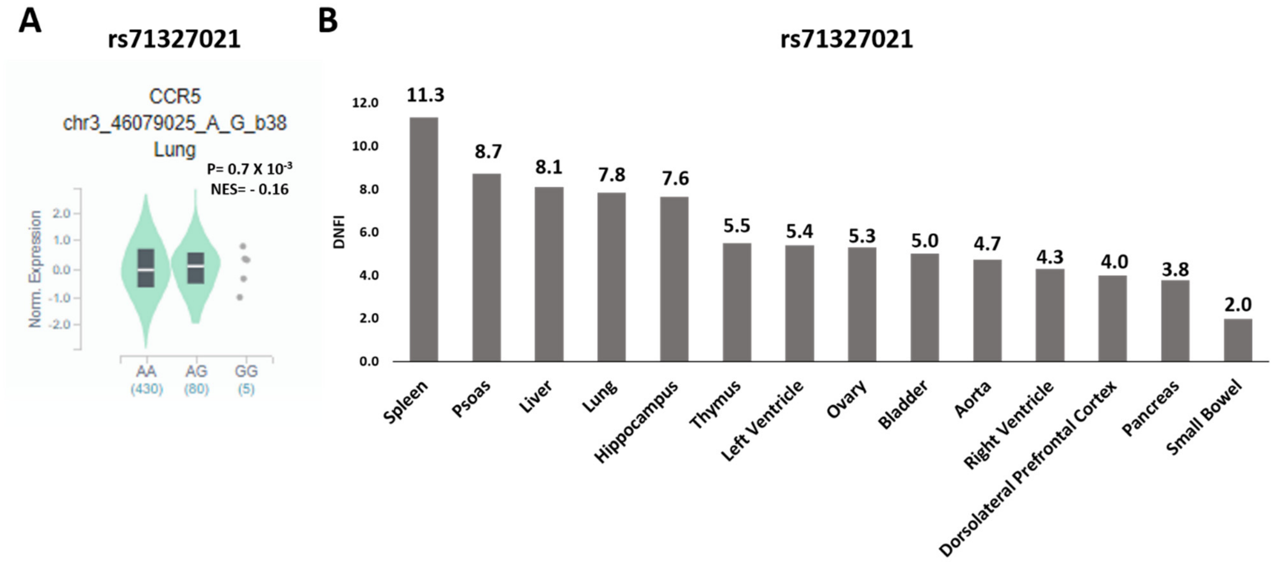

Figure S4. Minor allele of rs71327021 was associated with low expression of *CCR5* in lung but this last one is not tissue where interaction with *CCR5* is stronger. A) Violin plot shows the effect of eQTLs SNP rs71327021 on *CCR5* expression in lung. B) Hi-C data show interaction between rs71327021 and *CCR5* in all tissue. NES: normalized effect size.
